# Biochemical and Structural Characterisation of a Bacterial Lactoperoxidase

**DOI:** 10.1002/cbic.202400713

**Published:** 2024-11-29

**Authors:** Ognjen Pećanac, Caterina Martin, Simone Savino, Henriette J. Rozeboom, Marco W. Fraaije, Nikola Lončar

**Affiliations:** ^1^ GECCO Biotech Zernikepark 6 9747 AN Groningen The Netherlands; ^2^ Molecular Enzymology Group University of Groningen Nijenborgh 3 9747 AG Groningen The Netherlands

**Keywords:** biocatalysis, lactoperoxidase, bacterial, peroxidase, heme

## Abstract

Peroxidases belong to a group of enzymes that are widely found in animals, plants and microorganisms. These enzymes are effective biocatalysts for a wide range of oxidations on various substrates. This work presents a biochemical and structural characterization of a novel heme‐containing peroxidase from *Cyanobacterium sp*. TDX16, CyanoPOX. This cyanobacterial enzyme was successfully overexpressed in *Escherichia coli* as a soluble, heme‐containing monomeric enzyme. Although CyanoPOX shares relatively low sequence identity (37 %) with bovine lactoperoxidase, it displays comparable biochemical properties. CyanoPOX is most stable and active in slightly acidic conditions (pH 6–6.5) and moderately thermostable (melting temperature around 48 °C). Several compounds that are typical substrates for mammalian lactoperoxidases were tested to establish the catalytic potential of CyanoPOX. Potassium iodide showed the highest catalytic efficiency (126 mM^−1^ s^−1^), while various aromatic compounds were also readily converted. Structural elucidation of CyanoPOX confirmed the presence of a non‐covalently bound *b*‐type heme cofactor that is situated in the central core of the protein. Except for a highly similar overall structure, CyanoPOX also has a conserved active site pocket when compared with mammalian lactoperoxidases. Due to its catalytic properties and high expression in a bacterial host, this newly discovered peroxidase shows promise for applications.

## Graphical abstract

This study presents the biochemical and structural characterization of CyanoPOX, a heme‐containing peroxidase from *Cyanobacterium sp*. TDX16. Expressed in *E. coli*, the enzyme exhibits properties similar to bovine lactoperoxidase despite low sequence identity. CyanoPOX demonstrates optimal activity in slightly acidic conditions and efficiently oxidizes various substrates. Structural analysis reveals a non‐covalently bound b‐type heme cofactor and a conserved active site pocket.

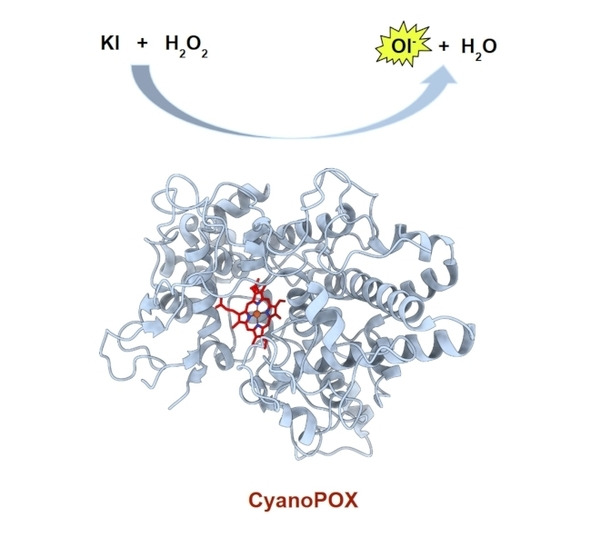

## Introduction

Peroxidases (EC 1.11.1.X) are peroxide‐driven oxidative enzymes that are ubiquitous in nature.^[1]^ These enzymes employ various peroxides (ROOH) as electron acceptors to catalyze numerous oxidation reactions. Peroxidases reduce hydrogen peroxide (H_2_O_2_) to water, along with simultaneous one‐ and/or two‐electron oxidations of a wide range of substrates.^[2]^ Most peroxidases utilize a tightly bound heme as cofactor.^[3]^ Several distinct major families can be distinguished for heme‐containing peroxidases which include (i) mammalian heme peroxidases, (ii) non‐animal heme peroxidases, and (iii) the microbial DyP‐type peroxidases.[^4^, ^5^, ^6^] The mammalian heme peroxidase family includes several well studied enzymes: lactoperoxidase (LPO), thyroid peroxidase (TPO), myeloperoxidase (MPO) and eosinophil peroxidase (EPO).^[7]^ These mammalian enzymes exhibit functional homology with heme‐containing peroxidases from other classes but differ vastly in their mechanism of ligand binding as well as in their primary and tertiary structures.[^2^, ^7^] Their unique features are also reflected in the binding mode of the heme cofactor. The heme in mammalian peroxidases is often covalently bonded to the protein,^[8]^ while this is not the case for peroxidases from other classes.[^9^, ^10^] LPO (EC 1.11.1.7) is a peroxidase that is known from its essential role in mammals. It is secreted into milk and in other organs and serves as an antimicrobial agent. Mammalian LPOs are glycosylated monomeric hemoenzymes of around 700 amino acids in length. They harbor a calcium binding site and a heme as prosthetic group that is located deep within the protein structure and which is covalently bound to one or two glutamate/aspartate residues via ester bonds.[^4^, ^11^] The calcium binding site is essential for peroxidase activity. Mammalian LPOs are known for their ability to catalyze the H_2_O_2_‐mediated oxidation of halides and pseudohalides to hypohalous and hypothiocyanous acids.[^12^, ^13^] Products of this enzymatic reaction are strong oxidizing agents that are toxic to pathogens, making LPOs a vital component of the host defense system.^[14]^ Given LPO's wide‐ranging antibacterial efficacy against pathogenic bacteria, its application as a natural food preservative has emerged.^[15]^ For example, LPO offers applications within the dairy industry for prolonging the shelf life of pasteurized milk.^[16]^ In addition to its potential use in food preservation, LPO is also considered in personal care products as it stands out in providing protection against dental caries.[^17^, ^18^]

This work presents the characterization of a newly discovered lactoperoxidase from the bacterium *Cyanobacterium* sp. TDX16. Intriguingly, the sequence of this bacterial LPO is only slightly similar to those of well‐studied mammalian LPOs. This bacterial homologue, named CyanoPOX, was overexpressed in *E. coli* as a soluble and active enzyme. CyanoPOX showed good pH and temperature tolerance and displayed activity with a set of substrates.[^19^, ^20^, ^21^] As mammalian LPOs are difficult to produce as recombinant proteins, this robust and easily expressed bacterial LPO may enable new LPO‐based applications.

## Results and Discussion

### Identification of CyanoPOX

Mammalian peroxidases are notoriously difficult to express as recombinant proteins. This may be due to the structural complexity of glycosylation and calcium binding, preventing proper folding in commonly used microbial expression hosts. With the goal to explore bacterial genomes for LPO homologs, bovine LPO (XP_024835317.1) was used in a pBLAST search (NCBI). This resulted in the identification of a predicted protein sequence (556 residues) from a gene of *Cyanobacterium* sp. TDX16 that shared 37 % sequence identity. It was decided to use a synthetic gene for expressing this bacterial protein, equipped with a His‐tag, in *E. coli*.

### Expression and Characteristics of CyanoPOX

**Figure 1:**
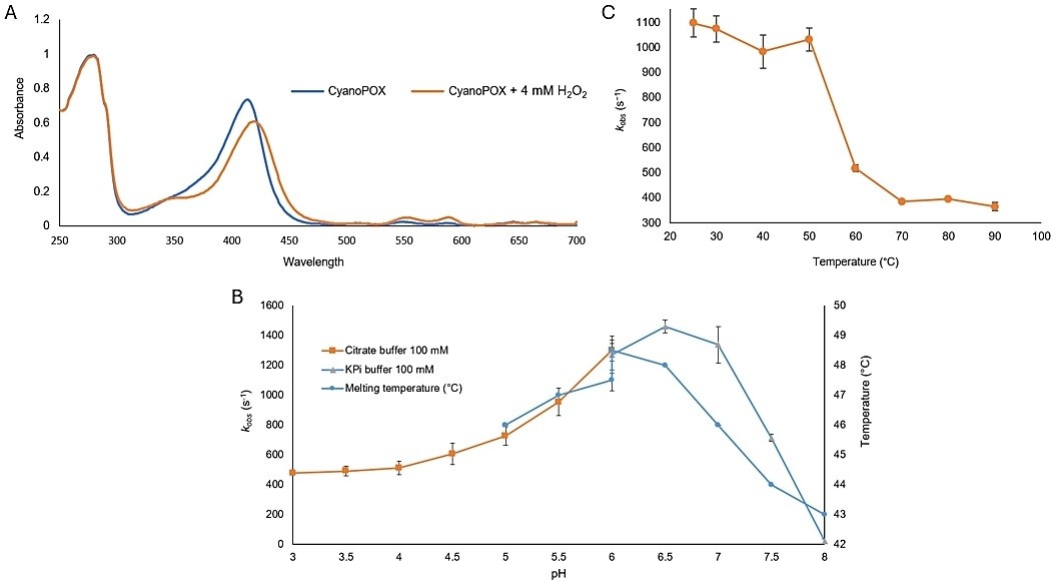
Characteristics of CyanoPOX. (A) UV‐visible spectrum of CyanoPOX incubated with 4 mM H_2_O_2_ for 30 minutes and without H_2_O_2_. (B) pH dependence of CyanoPOX activity and thermal stability. 40 mM KI, 0.5 mM H_2_O_2_ and 0.4 nM of purified CyanoPOX were used to measure CyanoPOX activity. The melting temperatures of CyanoPOX were measured in different 100 mM citrate and KPi buffers. No Tm values were obtained for the conditions in the moderately acidic pH range (3–4.5). (C) Effect of pre‐incubation temperature on CyanoPOX activity. 40 mM KI, 0.5 mM H_2_O_2_ and 0.4 nM of purified CyanoPOX in 50 mM KPi, pH 7.0 were used to measure CyanoPOX activity after 15 min pre‐incubations at defined temperatures.

Overexpression of the protein in *E. coli* NEB10β and subsequent purification yielded about 80 mg of red‐colored protein from 1 L of culture. SDS‐PAGE analysis revealed a distinct protein band at around 55 kDa (Figure S1). The observed molecular weight of this protein corresponded to the expected molecular weight of His‐CyanoPOX (calculated: 58 kDa). The UV‐visible absorbance spectrum of purified CyanoPOX (Figure 1A) exhibited maxima at 412 and 280 nm, which are characteristic for heme‐containing proteins. Also, the so‐called visible bands typical for hemoproteins were present in the 500–700 nm range. The intensity of the Soret band at 412 nm was slightly lower compared with the absorbance at 280 nm, resulting in a R_z_ value of 0.75 (A_412_/A_280_). R_z_ values for LPOs are typically close to 1 which may suggest that the CyanoPOX was not fully loaded with the heme cofactor. Incubation of CyanoPOX with 4.0 mM H_2_O_2_ for 30 minutes, resulted in a shift of the Soret band from 413 nm to 419 nm and a decrease in intensity (Figure 1A). These observations indicated that CyanoPOX is reactive with hydrogen peroxide.

### Stability of CyanoPOX

The ThermoFluor method was used to study the thermostability of CyanoPOX (Figure 1B). CyanoPOX demonstrated the highest thermostability in slightly acidic conditions. The highest T_m_ values (47–49 °C) were obtained at pH 6.0–6.5. Acidic conditions (pH <4.5) appeared to be too harsh for the enzyme as no T_m_ values could be obtained. Our results indicate that CyanoPOX exhibits a slightly higher thermostability when compared to bovine LPO, as the mammalian heme‐containing peroxidase displayed a T_m_ value of 43.5 °C at pH 6.0. A comparable pH‐dependent profile was observed for enzymatic activity (Figure 1B). CyanoPOX exhibited the highest activity at pH 6.5. These properties depict resemblance with the data reported for bovine LPO.^[19]^ CyanoPOX showed to retain activity upon 15 min incubations up to 50 °C (Figure 1C). This is in line with the observed melting temperature and shows that CyanoPOX is a moderately thermostable enzyme.

### Steady State Kinetics

The CyanoPOX substrate profile was evaluated to examine whether it shares similarities to mammalian LPOs. Steady state kinetic parameters were successfully obtained for several substrates (Table 1 and Figure S2). Initial rates of the reactions were acquired and were fitted using the Michaelis‐Menten formula. CyanoPOX demonstrated the highest activity with potassium iodide (KI) (*k*_cat_=885 s^−1^) and a relatively low K_M_ value (7.0 mM). A bright yellow color appeared when using this substrate, indicating the formation of hypoiodite (IO^−^). Furtmüller et al.^[22]^ report a second order rate constant (1.2×10^8^ M^−1^ s^−1^) for bovine LPO with iodide (I^−^). Bovine LPO from Sigma‐Aldrich was tested for activity with KI using the same conditions as CyanoPOX. Notably, CyanoPOX exhibited an 8‐fold higher catalytic efficiency (126 mM^−1^ s^−1^) compared to bovine LPO (15 mM^−1^ s^−1^), underscoring its efficiency for this specific reaction. CyanoPOX also showed good activity with other substrates that are typically accepted by mammalian LPOs.[^19^, ^20^, ^21^] Guaiacol and ABTS showed somewhat lower but still high *k*_cat_ values (135 and 570 s^−1^). However, the catalytic efficiencies for these two substrates are relatively low due to high *K*_M_ values. Guaiacol was also tested with bovine LPO using the same conditions as used for CyanoPOX, with results showing that CyanoPOX exhibited a 4‐fold higher catalytic efficiency (2 mM^−1^ s^−1^) compared to bovine LPO (0.5 mM^−1^ s^−1^). The high *K*_M_ value for ABTS seems to be specific for CyanoPOX when compared with the findings reported by Ozdemir et al. for bovine LPO.^[23]^ Furthermore, 2,6‐DMP exhibited a somewhat low *k*_cat_ value (620 s^−1^) when compared to KI. Due to the low K_M_ value (35 mM) for 2,6‐DMP compared to ABTS and guaiacol, 2,6‐DMP has emerged as the second‐best performing substrate after KI. Having an insight into the kinetic parameters of H_2_O_2_ for peroxidases is crucial for understanding its enzymatic activity and substrate affinity. CyanoPOX displays a *K*_M_ value of 0.48 mM for H_2_O_2_ which closely resembles the value (0.41 mM) reported for the bovine LPO by Burec et al.^[24]^

**Table 1:** Steady‐state kinetic parameters of CyanoPOX and bovine LPO. Reactions were performed at different enzyme concentrations, adjusted to fit a specific substrate. Activity was assessed by monitoring the formation of oxidized products from the substrates at different wavelengths.

Enzyme	Substrate	*k*_cat_ (s^−1^)	*K*_M_ (mM)	*k*_cat_/*K*_M_ (mM^−1^ s^−1^)
CyanoPOX	KI^[b]^	885	7	126
	2,6‐DMP^[b]^	620	35	18
	guaiacol^[b]^	135	59	2
	ABTS^[a]^	570	150	3.7
	H_2_O_2_^[c]^	1390	0.48	2900
bovine LPO	KI^[b]^	206	14	15
	guaiacol^[b]^	10	22	0.5

[a] Reaction was performed in 50 mM KPi, 150 mM NaCl, pH 5.0. [b] Reaction was performed in 50 mM KPi, 150 mM NaCl, pH 7.0. [c] Kinetic parameters for H_2_O_2_ were obtained through enzymatic activity analysis with KI at different H_2_O_2_ concentrations.

### Structural Elucidation of CyanoPOX

**Figure 2:**
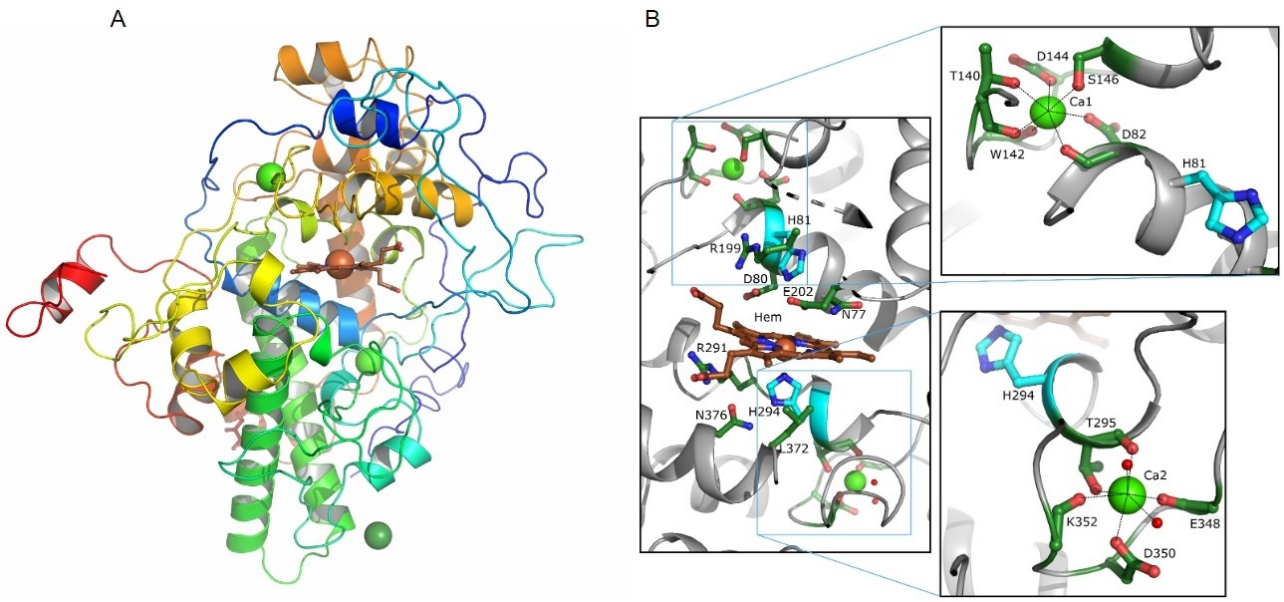
Structural features of CyanoPOX. (A) Three‐dimensional crystal structure of CyanoPOX. Ribbon diagram of the CyanoPOX molecule in rainbow colors (blue is N‐terminus to red C‐terminus). The heme cofactor is shown in sticks (represented with carbon in brown, oxygen in red, nitrogen in blue and the iron atom as brown‐orange sphere). Ca^2+^ ions are depicted in green and the Mg^2+^ ion in dark green. (B) Zoomed view of the active site of CyanoPOX and the calcium binding sites. Active site residues are shown as cyan sticks. Other important residues are depicted in forest green. The inset zooms into the calcium binding sites showing ligating residues and metal‐ligand bonds as black dashed lines.

For obtaining more detailed insights into the structural and catalytic properties of CyanoPOX, the enzyme was crystallized. The crystal structure of CyanoPOX with a bound heme cofactor was determined at 1.67 Å resolution in the monoclinic space group P2_1_ with one molecule in the asymmetric unit (Figure 2A). Size exclusion chromatography with subsequent DLS analysis indicated that CyanoPOX is monomeric in solution, with estimated molecular weights of 50 kDa and 34 kDa, respectively. Also, CyanoPOX in the crystal structure seems monomeric, with a size of 55×50×44 Å (Figure 2A). The closest structural homologues of CyanoPOX determined by a DALI search is LPO from *Dictyostelium discoideum* (Z_score_=48.9, 36 % identical on residue level, root‐mean‐square deviation of 1.8 Å, (PDB 6ERC).^[25]^ There is also structural homology with LPOs from yak (7DE5),^[26]^ water buffalo (3ERH),^[27]^ sheep (7VE3),^[28]^ goat (5FF1),^[29]^ bovine (7DN6)^[30]^ and the Human Myeloperoxidase (MPO) (1CXP),^[31]^ all with ~40 % sequence identity, Zscores of 34–46 and rmsd values of ~2.0 Å. The CyanoPOX structure includes residues 1–525. The three C‐terminal residues were not visible in electron density. The secondary structure of CyanoPOX is largely α‐helical and starts (according to DSSP) with H1(Ala48‐Val51), H2(Trp71‐Leu83), H2b(Ser146‐Tyr149), H3(Met153‐Leu159), H5(Ser204‐Ala227), H6(Gly233‐Leu261, Pro258 conserved), distorted H8(Asn283‐Met296), H9(Asn326‐Asn330), H10(Ala335‐Leu341), H11(Asp355‐Asn359), H12(Leu372‐Asp382), H13(Tyr388‐Met394), 3_10_ H14(Phe403‐Asp405), H15 (Pro410‐Tyr420), H16(Leu428‐Ala434), H17(Glu445‐Asp460), H18(Asp470‐Val473), H19(Pro476‐Gln483), H20(Leu487‐Arg493) and hydrophobic H21(Thr512‐Leu516). The secondary structural α‐helix numbering is according to Sharma et al.^[7]^ for LPOs. H2a (in LPO) and H2b (in CyanoPOX) are situated at a slightly different position and are therefore named differently. The short H4, situated on the surface of LPO, is missing in CyanoPOX. Furthermore, six short β‐strands, B1(Ile97‐Val99), B2(Thr115‐Ile116), B3(Thr300‐Ile301), B4(Leu316‐Ala317), B5(Phe360‐Leu361) and B6(Gly369‐Leu370) are present. The sequence of CyanoPOX is shorter than that of mammalian peroxidases. These enzymes contain a ~20 residue longer N‐terminus, an insert before H1, an inserted H4 and a ~10 residue longer C‐terminus. The cores of the enzymes overlap well, although the Dictyostelium peroxidase exhibits an excursion in the H2a/H2b region. Unlike eukaryotic peroxidases, CyanoPOX lacks disulfide bridges and N‐glycosylation sites. Interestingly, the last 22 residues at the C‐terminus of the CyanoPOX structure have a PEP‐CTERM protein‐sorting domain motif.^[32]^ It consists of a highly conserved Pro‐Glu‐Pro triad (residues 508–510) followed by a hydrophobic α‐helix (Thr512‐Leu513‐Gly514‐Leu515‐Leu516‐Met517) and finally a positively charged segment (524 till C‐terminus). It is believed that PEP‐CTERM forms a protein export sorting system and may be the recognition sequence for protein‐processing functions that could include protein modification, cleavage, sorting, and attachment.^[33]^ The six‐residue α‐helix within the motif is predicted to be transmembrane, but its length is insufficient to span the membrane, typically requiring 18 or more residues. To our knowledge this is the first time this motif has been observed in a crystal structure. A non‐covalently bound *b*‐type heme cofactor is situated in the central core of the CyanoPOX structure. The occupancy of the heme group was refined to 75 %. On the proximal heme site the conserved residues, His294, Leu372, Asn376 and Arg291 are situated. His81 is identified as the distal heme ligand (Figure 2B). Other residues close to the distal site of the porphyrin ring are Asn77 and Arg199. One of the propionate chains of the heme has salt bridges with the side chains of Arg291 and Arg379. The other propionate chain interacts via hydrogen bonds with Asp84 and backbone nitrogen of Thr86. The covalent bonds with the methyl groups of the heme observed in mammalian peroxidases to Asp80 and Glu202 (CyanoPOX numbering), through two ester bonds between OD2‐Asp and heme atom CMD, and OE2‐Glu and heme atom CMB, are absent, but the residues nevertheless remain. Potentially, the two heme‐to‐protein ester bonds have not been formed as they are derived from a hydrogen peroxide‐mediated post‐translational modification,^[34]^ as CyanoPOX did not undergo a peroxide treatment during purification/crystallization. The active site located in the distal heme cavity is easily accessed by the solvent. The hydrophobic entrance is large, about 11 Å in diameter (Figure 2B). A conserved calcium ion is observed on the distal site of the heme (11 Å to His81). Its ligands are OD1 and the carbonyl oxygen of Asp82 (adjacent to His81), OG1 and the carbonyl oxygen of Thr140, OD1 of Asp144, OG of Ser146 and the carbonyl oxygen of Trp142. Another non‐conserved calcium ion (10 Å to His294) is liganded by the sidechain of Asp350, the carbonyl oxygen and OG1 of Thr295 (adjacent to His294), the carbonyl oxygens of Glu348 and Lys352, and two water molecules (Figure 2B). Having these calcium ions close to the active site indicates they are structurally and catalytically important. Furthermore, a potential magnesium ion is located close to Met162 and surrounded by 4 water molecules at short distances (2.1–2.2 Å).

## Conclusions

Peroxidases are industrially relevant enzymes because they catalyze bio‐oxidations on multiple substrates and find applications in different fields.[^1^, ^2^] Nevertheless some of the most studied peroxidase representatives are of a eukaryotic origin. This inspired us to explore the presence of bacterial LPO homologues, as this mammalian heme‐containing peroxidase is one of the most studied representatives of this family and is a biotechnologically relevant enzyme. We have successfully identified and characterized a bacterial LPO, CyanoPOX, which can be overexpressed in *E. coli* with a yield of 80 mg/L. We observed that its pH stability is similar to mammalian LPOs, being most active and stable at slightly acidic conditions (pH 6–6.5). The somewhat higher thermostability of CyanoPOX, when compared with bovine LPO, may result from some structural differences between CyanoPOX and bovine LPO. The bacterial homolog is significantly smaller (∼23 kDa), partly due to truncations in the C‐ and N‐termini, harbors no disulfide bonds, and is not glycosylated. These features may also explain why expression of CyanoPOX in a bacterial host is highly efficient, while mammalian LPO cannot be expressed that easily. Steady state kinetic analysis of CyanoPOX exhibits a similar substrate profile compared to LPO, with the highest activity towards KI. LPO is known for its ability to catalyze the reactions sufficiently after a heat treatment at high temperatures for a short time.^[19]^ In this work we showed that CyanoPOX maintains high catalytic activity up to 50 °C, after which a steep decrease in activity is observed. This opens the possibility for additional research aimed at enhancing both the catalytic properties and stability.

## Experimental Section

### Chemicals and Materials

The synthetic gene encoding for CyanoPOX was ordered at IDT (Integrated DNA Technologies). Bovine LPO and chemicals were purchased from Sigma‐Aldrich and Fluorochem.

### Cloning, Expression, Purification and Characterization of CyanoPOX

With the assistance of IDT tools, we codon optimized the sequence (Table S1) that encodes the selected candidate enzyme to enable its expression in *E. coli*. Golden Gate methodology^[35]^ was used for cloning the synthetic gene in pBAD His vector (ampicillin resistance). pBAD SUMO vector was created similarly and used only for crystallization purposes. 5 μg of the obtained PCR mixture was added to 50 μL RbCl_2_ competent NEB10β *E. coli* cells. Following an incubation period of 30 min on ice, the cells were heat shocked for 45 seconds at 42 °C and placed on ice once more for another 3 minutes. 500 μL of SOC media was used to recover the cells at 37 °C for 45 min. Overnight cultures were prepared in 5 mL LB medium containing 100 μg/mL amp and incubated at 37 °C while being agitated at 135 rpm. 500 mL Terrific Broth medium supplemented with 100 μg/mL amp was used for the expression. Cultures were grown at 37 °C while being agitated at 135 rpm in a baffled flask until an OD_600_ reached ~0.6. Expression of CyanoPOX was commenced by adding L‐arabinose (0.02 % final concentration). Additionally, 5‐aminolevulinic acid (1 mM, final concentration) and iron sulfate (1 mM, final concentration) were introduced into the cultures to facilitate the biosynthesis of heme. Cultures were incubated at 17 °C, 135 rpm for 72 hours and then harvested by centrifugation (3700 rpm, 40 minutes, 4 °C). 33 mL lysis buffer (50 mM KPi, 150 mM NaCl, pH 7.5) was used to resuspend cell pellets which were subsequently disrupted by sonication (5 s on 5 s off, 10 minutes, 70 % amplitude). Supernatants were obtained by centrifuging at 12,000 rpm for 45 minutes at 4 °C and afterwards loaded onto Ni Sepharose gravity columns containing 3 mL resin. Columns were washed with a wash buffer (50 mM KPi, 150 mM NaCl, 20 mM imidazole, pH 7.5) equivalent to five times the column volume. The elution of proteins was performed using 3 mL of elution buffer (50 mM KPi, 150 mM NaCl, 500 mM imidazole, pH 7.5). PD10 columns were employed to substitute the elution buffer for a storage solution buffer (50 mM KPi, 150 mM NaCl, pH 7.5). Concentrations of proteins were measured using the NanoDrop ND 1000 UV‐Visible spectrophotometer (ϵ_412_=58.44 mM^−1^ cm^−1^).^[36]^ The UV‐Vis spectra of CyanoPOX were acquired in the range from 250–750 nm at 25 °C using a JASCO V‐660 spectrophotometer following the necessary enzyme dilution. Temperatures at which enzyme unfolding occurred were determined using the ThermoFluor method.^[37]^ Enzyme concentrations of 1 mg/mL were diluted 5‐fold in 100 mM citrate and KPi buffers with a range of pH values from 3.0 to 8.0. ThermoFluor assay was conducted starting at 20 °C and gradually increasing to 95 °C in 1 °C increments every 60 seconds, while monitoring the process with an RT‐PCR thermocycler (CFX96, Bio‐Rad). Retained activities upon thermal incubation of CyanoPOX were evaluated in a temperature range from 25–90 °C. The enzyme was incubated for 15 minutes in BioRAD T100 thermal cycler, followed by centrifugation using an Eppendorf microcentrifuge 5425 at 12,000 rpm for 2 minutes. Supernatants were collected for enzymatic activity analysis.

### Steady‐State Kinetics

Reactions using CyanoPOX and bovine LPO in 50 mM KPi, 150 mM NaCl, pH 7.0 were carried out at 25 °C using ABTS, KI, guaiacol and 2,6‐DMP as substrates at different concentrations. Enzyme concentration was adjusted to fit a specific substrate in order to be able to follow the enzyme kinetic in the linear range, while 0.5 mM H_2_O_2_ was used in all of the reactions. The pH optimum for CyanoPOX and activity measurements upon thermal incubation were determined through enzymatic activity analysis with KI at 25 °C. Reactions were performed with 40 mM KI, 0.5 mM H_2_O_2_ and 0.4 nM of purified CyanoPOX. Kinetic parameters were determined using 50 mM KPi, pH 7.0 and 0.4 nM of purified CyanoPOX. Substrate concentrations were varied and used in combination of 0.5 mM of H_2_O_2_ (when varying the substrate concentration) or 40 mM KI (when varying the hydrogen peroxide). Formation of oxidized products from the substrates was monitored (ABTS ϵ_420_=36.0 mM^−1^ cm^−1^;^[38]^ KI ϵ_350_=26.0 mM^−1^ cm^−1^;^[39]^ guaiacol ϵ_470_=26.6 mM^−1^ cm^−1[40]^ and 2,6‐DMP ϵ_469_=53.2 mM^−1^ cm^−1[41]^) on the BioTek Synergy HTX multi‐mode microplate reader. Initial rates of the reactions were obtained from the linear regions of the reaction curves. Data was processed using GraphPad Prism 6.05 (La Jolla, CA, USA).

### Crystallographic Studies

For crystallization experiments, another CyanoPOX expression construct was prepared for producing a SUMO‐tagged protein, with no change to the expression and purification methods previously employed. Purified His‐tagged SUMO‐CyanoPOX was concentrated with Amicon Ultra 30k 0.5 mL centrifuge filters to an adequate volume. The obtained sample was incubated overnight with His‐SUMO protease (1 mg/mL). CyanoPOX obtained after SUMO cleavage was further purified by gel filtration using a Superdex 200 HR10/30 column (Cytiva), equilibrated with 20 mM HEPES buffer, 150 mM NaCl, pH 7.3 on an ÄKTA explorer system with wavelengths set at 280, 254 and 412 nm. Tawny brown colored CyanoPOX fractions were pooled and concentrated to 13.7 mg/mL using an Ultracel‐30 K filter unit (Millipore). Dynamic light scattering (DLS) experiments were performed using a DynaPro MS800TC instrument (Wyatt Technology Corporation) at 294 K. DLS data were processed and analyzed with Dynamics software. Initial sitting‐drop crystallization screening was performed using a Mosquito crystallization robot (STP Labtech) in 96‐well MRC2 plates (Swissci). Crystals were grown from 0.1 M MES buffer pH 6.0 and 20 % PEG6000 supplied with 0.2 M magnesium chloride or 0.2 M calcium chloride. Prior to data collection, crystals were briefly soaked in a cryoprotectant solution containing the crystallization solution supported with 20 % glycerol, and flash‐cooled in liquid nitrogen. X‐ray diffraction data were recorded at the MASSIF‐1 beamline at the ESRF, Grenoble.^[42]^ Automatic data processing, using the program autoPROC with anisotropic analysis,^[43]^ was performed at the ESRF. The crystals belonged to space group P2_1_ with cell dimensions of a=52.7, b=72.5, c=56.2 Å and β=95.9°. The VM is 1.9 Å^3^/Da with a solvent content of 34 %, indicating a tightly packed crystal.^[44]^ The structure of CyanoPOX could be determined by molecular replacement using Phaser^[45]^ with an AlphaFold2^[46]^ (ColabFold) model. The asymmetric unit contained 1 monomer of 57.9 kDa. Refinement and model building was done using the programs Coot and REFMAC5.[^45^, ^47^] The heme cofactor was built in the F_o_–F_c_ electron density map and occupancy refinement was done with phenix.refine.^[48]^ The quality of the model was analyzed with PDB_REDO and MolProbity.^[45]^ PyMOL (Schrödinger) was used for figure preparation. Data collection statistics and refinement details are recorded in (Table S2). Atomic coordinates and experimental structure factor amplitudes were deposited in the Protein Data Bank PDB number 8S6C.

## Conflict of Interests

M. W. F. and N. L. are named inventors on a patent filing on the production and use of a bacterial lactoperoxidase.

1

## Supporting information

As a service to our authors and readers, this journal provides supporting information supplied by the authors. Such materials are peer reviewed and may be re‐organized for online delivery, but are not copy‐edited or typeset. Technical support issues arising from supporting information (other than missing files) should be addressed to the authors.

Supporting Information

## Data Availability

The data that support the findings of this study are available from the corresponding author upon reasonable request.

## References

[1] F. K. de Oliveira , L. O. Santos , J. G. Buffon , Food Res. Int. 2021, 143, 110266. 33992367 10.1016/j.foodres.2021.110266

[2] M. Zamocky , C. Jakopitsch , P. G. Furtmüller , C. Dunand , C. Obinger , Proteins: Struct. Funct. Bioinformatics. 2008, 72, 589–605. 10.1002/prot.21950 18247411

[3] D. Koua , L. Cerutti , L. Falquet , C. J. Sigrist , G. Theiler , N. Hulo , C. Dunand , Nucleic Acids Res. 2009, 37, 261–266. 10.1093/nar/gkn680 PMC2686439 18948296

[4] P. K. Singh , H. V. Sirohi , N. Iqbal , P. Tiwari , P. Kaur , S. Sharma , T. P. Singh , Biochim. Biophys. Acta (BBA) - Proteins Proteomics. 2017, 1865, 329–335. 27986533 10.1016/j.bbapap.2016.12.006

[5] N. Fawal , Q. Li , B. Savelli , M. Brette , G. Passaia , M. Fabre , C. Mathe , C. Dunand , Nucleic Acids Res. 2012, 41, 441–444. 10.1093/nar/gks1083 PMC3531118 23180785

[6] M. Zamocky , S. Hofbauer , I. Schaffner , B. Gasselhuber , A. Nicolussi , M. Soudi , K. F. Pirker , P. G. Furtmüller , C. Obinger , Arch. Biochem. Biophys. 2015, 574, 108–119. 25575902 10.1016/j.abb.2014.12.025 PMC4420034

[7] S. Sharma , A. K. Singh , S. Kaushik , M. Sinha , R. P. Singh , P. Sharma , H. Sirohi , P. Kaur , T. P. Singh , Int. J. Biochem. Mol. Biol. 2013, 4, 108. 24049667 PMC3776144

[8] C. Oxvig , A. R. Thomsen , M. T. Overgaard , E. S. Sørensen , P. Højrup , M. J. Bjerrum , G. J. Gleich , L. Sottrup-Jensen , J. Biol. Chem. 1999, 274, 16953–16958. 10358043 10.1074/jbc.274.24.16953

[9] D. J. Schuller , N. Ban , R. B. van Huystee , A. McPherson , T. L. Poulos , Structure 1996, 4, 311–321. 8805539 10.1016/s0969-2126(96)00035-4

[10] N. Kunishima , K. Fukuyama , H. Matsubara , H. Hatanaka , Y. Shibano , T. Amachi , J. Mol. Biol. 1994, 235, 331–344. 8289254 10.1016/s0022-2836(05)80037-3

[11] K. Shin , H. Hayasawa , B. Lönnerdal , Biochem. Biophys. Res. Commun. 2001, 281, 1024–1029. 11237766 10.1006/bbrc.2001.4448

[12] J. E. Harrison , J. Schultz , J. Biol. Chem. 1976, 251, 1371–1374. 176150

[13] R. Wever , W. M. Kast , J. H. Kasinoedin , R. Boelens , Biochim. Biophys. Acta (BBA) - Protein Struct. Mol. Enzymol. 1982, 709, 212–219. 10.1016/0167-4838(82)90463-0 6295491

[14] H. S. Garcia , Á. Lopez-Hernandez , C. G. Hill , Comprehensive Biotechnol. 2011, 4, 567–574.

[15] Š. Gruden , J. Oberčkal , B. B. Matijašić , N. P. Ulrih , Int. Dairy J. 2023, 138, 105537.

[16] E. Seifu , E. M. Buys , E. F. Donkin , Trends Food Sci. Technol. 2005, 16, 137–154.

[17] M. Magacz , K. Kędziora , J. Sapa , W. Krzyściak , Int. J. Mol. Sci. 2019, 20, 1443. 30901933 10.3390/ijms20061443 PMC6472183

[18] R. K. Gudipaneni , V. Kumar , G. Jesudass , S. Peddengatagari , Y. Duddu , J. Clin. Diagn. Res. 2014, 8, 18–20. 10.7860/JCDR/2014/8161.4232 PMC4064914 24959510

[19] B. Ozer, in *Encyclopedia of Food Microbiology*., *2nd edn*. (Eds.: C. A. Batt, R. K. Robinson), Amsterdam, The Netherlands, **2014**, pp. 930–935.

[20] A. Kalluri , M. K. Puglia , M. Malhotra , C. V. Kumar , Methods Enzymol. 2020, 630, 407–430. 31931996 10.1016/bs.mie.2019.11.010

[21] S. Y. Okazaki , Y. Uchimura , M. Goto , S. Furusaki , Biochem. Eng. J. 2000, 6, 103–107. 10959083 10.1016/s1369-703x(00)00078-4

[22] P. G. Furtmüller , W. Jantschko , G. Regelsberger , C. Jakopitsch , J. Arnhold , C. Obinger , Biochem. 2002, 41, 11895–11900. 12269834 10.1021/bi026326x

[23] H. Ozdemir , I. Aygul , O. I. Küfrevioglu , Preparative Biochem. Biotechnol. 2001, 31, 125–134. 10.1081/PB-100103378 11426700

[24] B. Burec , M. Jušić , P. Mildner , Croat. Chem. Acta. 1963, 35, 153–159.

[25] A. Nicolussi , J. D. Dunn , G. Mlynek , M. Bellei , M. Zamocky , G. Battistuzzi , K. Djinović-Carugo , P. G. Furtmüller , T. Soldati , C. Obinger , J. Biol. Chem. 2018, 293, 1330–1345. 29242189 10.1074/jbc.RA117.000463 PMC5787809

[26] V. Viswanathan , C. Rani , N. Ahmad , P. K. Singh , P. Sharma , P. Kaur , S. Sharma , T. P. Singh , The Protein J. 2021, 40, 8–18. 33389415 10.1007/s10930-020-09957-2

[27] I. A. Sheikh , A. K. Singh , N. Singh , M. Sinha , S. B. Singh , A. Bhushan , P. Kaur , A. Srinivasan , S. Sharma , T. P. Singh , J. Biol. Chem. 2009, 284, 14849–14856. 19339248 10.1074/jbc.M807644200 PMC2685666

[28] P. K. Singh , N. Ahmad , S. Yamini , R. P. Singh , A. K. Singh , P. Sharma , M. L. Smith , S. Sharma , T. P. Singh , Protein Sci. 2022, 31, 384–395. 34761444 10.1002/pro.4230 PMC8819834

[29] R. P. Singh , A. Singh , H. V. Sirohi , A. K. Singh , P. Kaur , S. Sharma , T. P. Singh , FEBS Open Bio. 2016, 6, 640–650. 10.1002/2211-5463.12051 PMC4932444 27398304

[30] P. K. Singh , P. Sharma , A. Bhushan , P. Kaur , S. Sharma , T. P. Singh , J. Inorg. Biochem. 2021, 220, 111461. 33882424 10.1016/j.jinorgbio.2021.111461

[31] T. J. Fiedler , C. A. Davey , R. E. Fenna , J. Biol. Chem. 2000, 275, 11964–11971. 10766826 10.1074/jbc.275.16.11964

[32] D. H. Haft , I. T. Paulsen , N. Ward , J. D. Seleng , BMC Biol. 2006, 4, 29. 16930487 10.1186/1741-7007-4-29 PMC1569441

[33] D. H. Haft , S. H. Payne , J. D. Selengut , J. Bacteriol. 2012, 194, 36–48. 22037399 10.1128/JB.06026-11 PMC3256604

[34] A. K. Singh , N. Singh , S. Sharma , S. B. Singh , P. Kaur , A. Bhushan , A. Srinivasan , T. P. Singh , J. Mol. Biol. 2008, 376, 1060–1075. 18191143 10.1016/j.jmb.2007.12.012

[35] C.Engler, S. Marillonnet in *Methods in Molecular Biology, Vol. 1116* (Eds.: S. Valla, L. Rahmi), Totowa, New Jersey, **2013**, pp. 119–131.

[36] C. Deniau , R. Gilli , N. Izadi-Pruneyre , S. Létoffé , M. Delepierre , C. Wandersman , C. Briand , A. Lecroisey , Biochem. 2003, 42, 10627–10633. 12962486 10.1021/bi030015k

[37] M. D. Cummings , M. A. Farnum , M. I. Nelen , SLAS Discov. 2006, 11, 854–863. 10.1177/1087057106292746 16943390

[38] T. Kenzom , P. Srivastava , S. Mishra , Appl. Environ. Microbiol. 2014, 80, 7484–7495. 25261507 10.1128/AEM.02665-14 PMC4249244

[39] R. P. Ferrari , E. M. Ghibaudi , S. Traversa , E. Laurenti , L. De Gioia , M. Salona , J. Inorg. Biochem. 1997, 68, 17–26. 9379177 10.1016/s0162-0134(97)00003-2

[40] R. S. Koduri , M. Tien , J. Biol. Chem. 1995, 270, 22254–22258. 7673205 10.1074/jbc.270.38.22254

[41] E. Breslmayr , S. Daly , A. Požgajčić , H. Chang , C. Rezić , C. Oostenbrink , R. Ludwig , Biotechnol. Biofuels. 2019, 12, 1–12. 31827611 10.1186/s13068-019-1624-3 PMC6894463

[42] M. W. Bowler , D. Nurizzo , R. Barrett , A. Beteva , M. Bodin , H. Caserotto , S. Delagenière , F. Dobias , D. Flot , T. Giraud , N. Guichard , J. Synchrotron Radiat. 2015, 22, 1540–1547. 26524320 10.1107/S1600577515016604 PMC4629869

[43] C. Vonrhein , C. Flensburg , P. Keller , A. Sharff , O. Smart , W. Paciorek , T. Womack , G. Bricogne , Acta Crystallogr. Sect. D: Biol. Crystallogr. 2011, 67, 293–302. 21460447 10.1107/S0907444911007773 PMC3069744

[44] B. W. Matthews , J. Mol. Biol. 1968, 33, 491–497. 5700707 10.1016/0022-2836(68)90205-2

[45] J. Agirre , M. Atanasova , H. Bagdonas , C. B. Ballard , A. Baslé , J. Beilsten-Edmands , R. J. Borges , D. G. Brown , J. J. Burgos-Mármol , J. M. Berrisford , P. S. Bond , Acta Crystallogr. Sect. D: Struct. Biol. 2023, 79, 449–461. 37259835 10.1107/S2059798323003595 PMC10233625

[46] J. Jumper , R. Evans , A. Pritzel , T. Green , M. Figurnov , O. Ronneberger , K. Tunyasuvunakool , R. Bates , A. Žídek , A. Potapenko , A. Bridgland , Nature. 2021, 596, 583–589. 34265844 10.1038/s41586-021-03819-2 PMC8371605

[47] P. Emsley , B. Lohkamp , W. G. Scott , K. Cowtan , Acta Crystallogr. Sect. D: Biol. Crystallogr. 2010, 66, 486–501. 20383002 10.1107/S0907444910007493 PMC2852313

[48] D. Liebschner , P. V. Afonine , M. L. Baker , G. Bunkóczi , V. B. Chen , T. I. Croll , B. Hintze , L. W. Hung , S. Jain , A. J. McCoy , N. W. Moriarty , Acta Crystallogr. Sect. D: Struct. Biol. 2019, 75, 861–877. 31588918 10.1107/S2059798319011471 PMC6778852

