# Supplementary material for: Biochemical and Structural Characterisation of a Bacterial Lactoperoxidase
Source: Chembiochem. 2024 Nov 29;26(2):e202400713. doi: 10.1002/cbic.202400713 (PMC11776367; doi:10.1002/cbic.202400713)
Supplement: Supplementary file 1 — Supporting Information [file CBIC-26-e202400713-s001.pdf]

ChemBioChem

Supporting Information

Biochemical and Structural Characterisation of a Bacterial Lactoperoxidase

Ognjen Pećanac, Caterina Martin, Simone Savino, Henriette J. Rozeboom, Marco W. Fraaije,* and Nikola Lončar*

ChemBioChem

Supporting Information

Biochemical and Structural Characterisation of a Bacterial Lactoperoxidase

Ognjen Pećanac, Caterina Martin, Simone Savino, Henriette J. Rozeboom, Marco W. Fraaije and Nikola Lonča

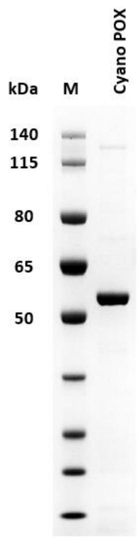

Figure S1. SDS-PAGE analysis of purified His_6-tagged CyanoPOX. M = marker.

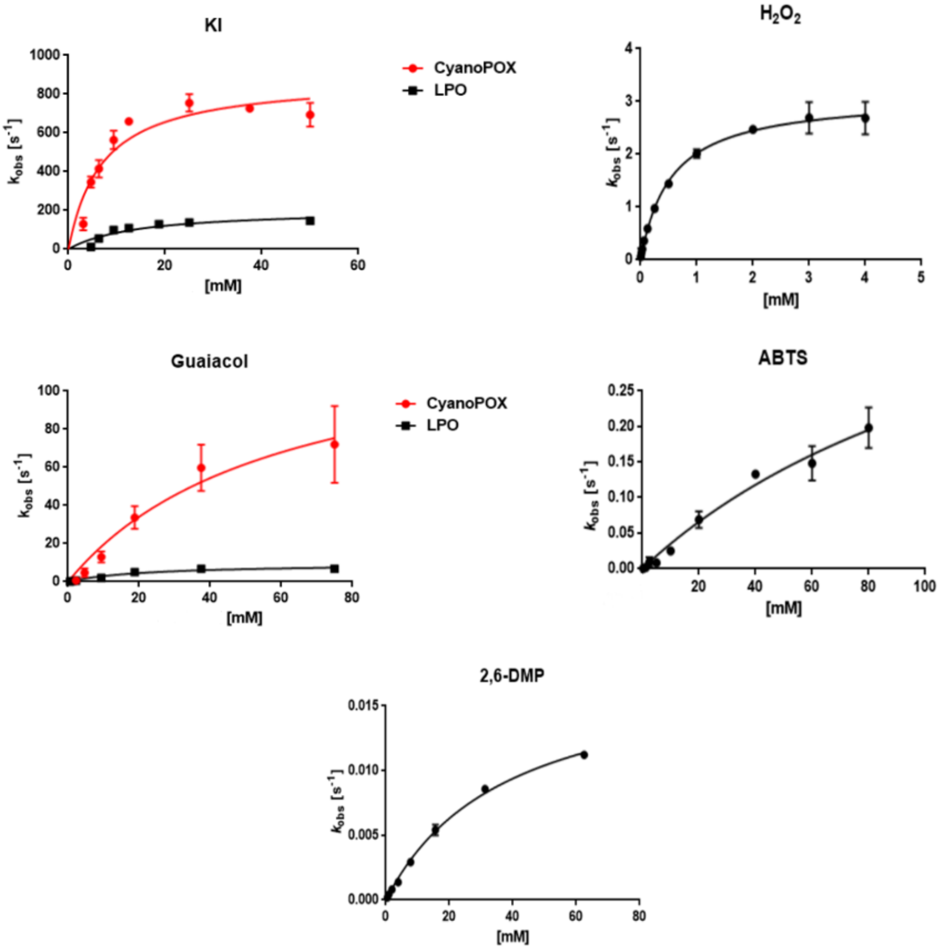

Figure S2. Michaelis-Menten kinetics for CyanoPOX and bovine LPO from Sigma-Aldrich on different substrates. Measurements were taken in duplicate.

Table S1. Sequence of *E. coli* codon optimized CyanoPOX gene.

Name of the gene	Sequence ‘3 - ‘5
CyanoPOX-His	ATGGGCAGCAGCCATCATCATCATCATCACGGCAGCGGCCTGGTGCCGCGCGGCAGCGCTGCACAGAAT<br>CGCACGATCGATGGGACGGATAACAATTTGAGTCATAACGTTTGGGGTTCAACAAACCAACATTTGGATCG<br>TGCAGGACCCGCGGCTTACGCGGATGGCATGTCCATGCCTGCCGGCGGATCTCGCCCTTCCGCTCGCGC<br>CGTATCGAATGGAATCGCCGCCCAGACTGGTTCAATGCTGAACGATCGCATGTTAAGCGATTGGGTTTGGC<br>AATGGGGGCAGTTCCTGGATCATGACTTAGATCTTACAGATGCGGCGTCCCCCGCTGAATCATTCCCGATC<br>CCTGTCCCCATGGGGGACCCCTTCTTCGACCCCTTCAACACAGGAACCCAGACCATCGGGCTTTCGCGCA<br>GCGCTTATGATCCTGCGACCGGCTCCGTAGATGCGCGCCAGCAAATGAATCAGATCACGTCCTGGATTGAT<br>GCTTCGAATGTGTATGGCAGCGACATGACGCGTGCCAACGCATTGCGTACTATGTCCGGAGGCCGTTTAG<br>CTACTAGTGCTGGTGATCTTTTACCCTTTAACACCGGTGGTCTTCCCAATGCTGGCGGCACAAGTCCTTCGT<br>TATTCCTGGCTGGTGATGTACGTAGCAATGAGCAATCTGGTTTGGCAGCGGTACACACATTATTTGTGCGC<br>GAGCACAACCGTCTTGCTGATCAGATTGCCGCCGCCAACCCGGGCATGGGAGATGAAGATATCTATCAGC<br>AGGCGCGTAAGATCGTCGGAGCTCAGATGCAGATTATTACATACAATGAATTTTTGCCAGCCCTGTTAGGTA<br>GCGCTGCGCCTTCGCCGATGAGTATTGGGTACGACGATAGCATTAATCCAAACATTATGAACGAGTTCGCT<br>AATGCGTGCTATCGCGTAGGACATACAATGCTTAGCCCAACGATCCTGCGCTTAGATAACGCGGGCAATGT<br>GATCCCCCACGGCAATTTGGCCTTACAGGATGCATTTTTTAATCCAAACCGCATTATCAACGAAGGTGGGAT<br>TGCTCCTATCTTGAAGGGTTTGGCAAGTCAAGCGATGCAAGAGATCGATAATAAAATCGTCGACGATGTAC<br>GCAATTTCTTATTCGGCCCTCCCGGTAGCGGAGGCTTAGATTTGGCTTCCTTGAATATCCAGCGTGGTCGC<br>GACCACGGGTTGCCCGATTATAATTCAACGCGCGTCATGATGGGATTAACCAGCGTCTCCAGCTTTGCGGA<br>TATTTCAAGCGACCCCGCAGTACAAGCAGCATTGATGTCACTTTACGGGACTGTGAATGATATCGACCTGT<br>GGGTAGGAGCCCTTGCTGAAGATCATTTAGCAGGTTCATCAGTTGGGGAGTTAATTGCGGCGGTTTTGGGC<br>GAACAGTTCACCCGTTTGCGTGATGGCGATCGCTATTGGTATGAACGTGATGATTTTTTCGTCAACAATCCA<br>TCGTTACTGGCTGAATTGCAGGCCACGCGTCTGAGTGATATTATTCGTCGTAACTCGGATATTACGAATATT<br>CAGGACAACGTATTCCTTATTCCGGAGCCGGCTACTTTGGGCCTTTTAATGTTCGGAGCAGCGTTTTTGCGT<br>AAACGTCGCTCG
CyanoPOX-His-SUMO	ATGGGCAGCAGCCATCATCATCATCATCACGGCAGCGGCCTGGTGCCGCGCGGCAGCGCTAGCATGTCG<br>GACTCAGAAGTCAATCAAGAAGCTAAGCCAGAGGTCAAGCCAGAAGTCAAGCCTGAGACTCACATCAATTT<br>AAAGGTGTCCGATGGATCTTCAGAGATCTTCTTCAAGATCAAAAAGACCACTCCTTTAAGAAGGCTGATGGA<br>AGCGTTCGCTAAAAGACAGGGTAAGGAAATGGACTCCTTAAGATTCTTGTACGACGGTATTAGAATTCAAGC<br>TGATCAGACCCCTGAAGATTTGGACATGGAGGATAACGATATTATTGAGGCTCACAGAGAACAGATTGGTG<br>GTGCACAGAATCGCACGATCGATGGGACGGATAACAATTTGAGTCATAACGTTTGGGGTTCAACAAACCAA<br>CATTTGGATCGTGCAGGACCCGCGGCTTACGCGGATGGCATGTCCATGCCTGCCGGCGGATCTCGCCCTT<br>CCGCTCGCGCCGTATCGAATGGAATCGCCGCCCAGACTGGTTCAATGCTGAACGATCGCATGTTAAGCGA<br>TTGGGTTTGGCAATGGGGGCAGTTCCTGGATCATGACTTAGATCTTACAGATGCGGCGTCCCCCGCTGAAT<br>CATTCCCGATCCCTGTCCCCATGGGGGACCCCTTCTTCGACCCCTTCAACACAGGAACCCAGACCATCGG<br>GCTTTCGCGCAGCGCTTATGATCCTGCGACCGGCTCCGTAGATGCGCGCCAGCAAATGAATCAGATCACG<br>TCCTGGATTGATGCTTCGAATGTGTATGGCAGCGACATGACGCGTGCCAACGCATTGCGTACTATGTCCGG<br>AGGCCGTTTAGCTACTAGTGCTGGTGATCTTTTACCCTTTAACACCGGTGGTCTTCCCAATGCTGGCGGCA<br>CAAGTCCTTCGTTATTCCTGGCTGGTGATGTACGTAGCAATGAGCAATCTGGTTTGGCAGCGGTACACACA<br>TTATTTGTGCGCGAGCACAACCGTCTTGCTGATCAGATTGCCGCCGCCAACCCGGGCATGGGAGATGAAG<br>ATATCTATCAGCAGGCGCGTAAGATCGTCGGAGCTCAGATGCAGATTATTACATACAATGAATTTTTGCCAG<br>CCCTGTTAGGTAGCGCTGCGCCTTCGCCGATGAGTATTGGGTACGACGATAGCATTAATCCAAACATTATG<br>AACGAGTTCGCTAATGCGTGCTATCGCGTAGGACATACAATGCTTAGCCCAACGATCCTGCGCTTAGATAA<br>CGCGGGCAATGTGATCCCCCACGGCAATTTGGCCTTACAGGATGCATTTTTTAATCCAAACCGCATTATCAA<br>CGAAGGTGGGATTGCTCCTATCTTGAAGGGTTTGGCAAGTCAAGCGATGCAAGAGATCGATAATAAAATCG<br>TCGACGATGTACGCAATTTCTTATTCGGCCCTCCCGGTAGCGGAGGCTTAGATTTGGCTTCCTTGAATATCC<br>AGCGTGGTCGCGACCACGGGTTGCCCGATTATAATTCAACGCGCGTCATGATGGGATTAACCAGCGTCTC<br>CAGCTTTGCGGATATTTCAAGCGACCCCGCAGTACAAGCAGCATTGATGTCACTTTACGGGACTGTGAATG<br>ATATCGACCTGTGGGTAGGAGCCCTTGCTGAAGATCATTTAGCAGGTTCATCAGTTGGGGAGTTAATTGCG<br>GCGGTTTTGGGCGAACAGTTCACCCGTTTGCGTGATGGCGATCGCTATTGGTATGAACGTGATGATTTTTT<br>CGTCAACAATCCATCGTTACTGGCTGAATTGCAGGCCACGCGTCTGAGTGATATTATTCGTCGTAACTCGG<br>ATATTACGAATATTCAGGACAACGTATTCCTTATTCCGGAGCCGGCTACTTTGGGCCTTTTAATGTTCGGAG<br>CAGCGTTTTTGCGTAAACGTCGCTCGTAA

Table S2. Crystallographic data collection and refinement statistics.

	CyanoPOX
Resolution range (Å)[a]	55.9 – 1.74 (1.78 - 1.74)
Cell dimensions (Å) a, b, c, α, β, γ	52.7, 72.5, 56.2, 90.0, 95.8, 90.0
Number of observations[a]	284521 (12328)
Number of unique reflections[a]	42540 (2171)
Completeness (%)[a]	97.9 (100.0)
Multiplicity[a]	6.7 (5.7)
$CC_{1/2}$[a]	0.997 (0.419)
Overall I/σ (I)[a]	9.2 (0.9)
R_{merge} (%)[a]	0.112 (1.828)
R_{pim} (%)[a]	0.047 (0.832)
R/ R_{free} (%)	15.1 / 18.8
Protein residues	525
Protein *B* value ($Å^2$)	29.0
Heme molecules	1
Heme *B* values ($Å^2$)	33.5
Glycerol molecules	1
Ca^{2+}/ Mg^{2+} ions	2/1
Water molecules	363
r.m.s.d. bonds (Å)	0.009
r.m.s.d. angles (°)	1.63
Ramachandran outliers	0.0
favored	97.7
Clashscore	5.3
Molprobity score	1.53
PDB accession ID	8S6C

[a] Values in parentheses are for the highest resolution shell.
